# Efficacy of the SOAR knee health program: protocol for a two-arm stepped-wedge randomized delayed-controlled trial

**DOI:** 10.1186/s12891-022-05019-z

**Published:** 2022-01-25

**Authors:** Jackie L. Whittaker, Linda K. Truong, Justin M. Losciale, Trish Silvester-Lee, Maxi Miciak, Andrea Pajkic, Christina Y. Le, Alison M. Hoens, Amber Mosewich, Michael A. Hunt, Linda C. Li, Ewa M. Roos

**Affiliations:** 1 Department of Physical Therapy, Faculty of Medicine, University of British Columbia, Vancouver, Canada; 2 Arthritis Research Canada, Vancouver, Canada; 3 Faculty of Rehabilitation Medicine, University of Alberta, Edmonton, Canada; 4 Faculty of Kinesiology, Sport and Recreation, University of Alberta, Edmonton, Canada; 5 Department of Musculoskeletal Function and Physiotherapy, University of Southern Denmark, Odense, Denmark

**Keywords:** Anterior cruciate ligament, Knee trauma, Post-traumatic osteoarthritis, Physiotherapy

## Abstract

**Background:**

Knee trauma permanently elevates one’s risk for knee osteoarthritis. Despite this, people at-risk of post-traumatic knee osteoarthritis rarely seek or receive care, and accessible and efficacious interventions to promote knee health after injury are lacking. Exercise can ameliorate some mechanisms and independent risk factors for osteoarthritis and, education and action-planning improve adherence to exercise and promote healthy behaviours.

**Methods:**

To assess the efficacy of a virtually-delivered, physiotherapist-guided exercise-based program (SOAR) to improve knee health in persons discharged from care after an activity-related knee injury, 70 people (16–35 years of age, 12–48 months post-injury) in Vancouver Canada will be recruited for a two-arm step-wedged assessor-blinded delayed-control randomized trial. Participants will be randomly allocated to receive the intervention immediately or after a 10-week delay. The program consists of 1) one-time Knee Camp (group education, 1:1 individualized exercise and activity goal-setting); 2) weekly individualized home-based exercise and activity program with tracking, and; 3) weekly 1:1 physiotherapy-guided action-planning with optional group exercise class. Outcomes will be measured at baseline, 9- (primary endpoint), and 18-weeks. The primary outcome is 9-week change in knee extension strength (normalized peak concentric torque; isokinetic dynamometer). Secondary outcomes include 9-week change in moderate-to-vigorous physical activity (accelerometer) and self-reported knee-related quality-of-life (Knee injury and OA Outcome Score subscale) and self-efficacy (Knee Self Efficacy Scale). Exploratory outcomes include 18-week change in primary and secondary outcomes, and 9- and 18- week change in other components of knee extensor and flexor muscle function, hop function, and self-reported symptoms, function, physical activity, social support, perceived self-care and kinesiophobia. Secondary study objectives will assess the feasibility of a future hybrid effectiveness-implementation trial protocol, determine the optimal intervention length, and explore stakeholder experiences.

**Discussion:**

This study will assess the efficacy of a novel, virtually-delivered, physiotherapist-guided exercise-based program to optimize knee health in persons at increased risk of osteoarthritis due to a past knee injury. Findings will provide valuable information to inform the management of osteoarthritis risk after knee trauma and the conduct of a future effectiveness-implementation trial.

**Trial registration:**

Clinicaltrials.gov reference: NTC04956393. Registered August 5, 2021, https://clinicaltrials.gov/ct2/show/NCT04956393?term=SOAR&cond=osteoarthritis&cntry=CA&city=Vancouver&draw=2&rank=1

**Supplementary Information:**

The online version contains supplementary material available at 10.1186/s12891-022-05019-z.

## Background

The Global Burden of Disease Study reports osteoarthritis (OA) as one of the fastest growing and burdensome conditions worldwide, [1] driven primarily by OA of the knee [2]. Given there is no cure for OA disease (articular and periarticular pathology), and only modestly effective treatments for OA illness (pain, disability, reduced quality-of-life; QoL), there is a desperate need for effective and accessible prevention interventions that strategically target at-risk populations [3].

Knee trauma is associated with a 6-fold increased risk of radiographic OA by 11 years, [4] and 6-fold increased risk of arthroplasty [5]. OA risk varies by injury type with cruciate ligament, meniscal, fracture, dislocation and collateral ligament injuries associated with 5-fold or higher risk [4]. Knee trauma is most prevalent in persons aged 16–35 years, and most commonly activity related [6]. Due to a relative young age at injury, people with knee trauma develop OA earlier compared to those without trauma, resulting in greater years lived with disability and reduced QoL.

The elevated risk for OA after trauma is driven by altered cartilage metabolism, [7] altered loading, [8] and inflammation [9]. We have also shown that youth with knee trauma up to 10-years previous have more independent OA risk factors (i.e., quadricep weakness, inactivity, adiposity) [10–12] than uninjured peers. Despite carrying these modifiable risk factors, people at-risk of OA after trauma are rarely aware of their risk, nor do they seek, or receive care to manage this risk [13, 14].

Exercise ameliorates several mechanisms (altered loading, [15] inflammation [16]) and independent risk factors (muscle weakness, [17] inactivity, [18] adiposity [19]) for OA. Despite this, the current standard of care after discharge from knee trauma treatment is no care, and the value of exercise-based activities to modify OA risk factors after trauma is unclear [20]. Given that knee trauma permanently elevates OA risk, strategies that enhance self-management, exercise adherence, and healthy lifestyles, such as informational support and action-planning are valuable adjuncts to exercise [21–23].

SOAR (Stop OsteoARthritis) is a virtually-delivered, physiotherapist (PT)-guided knee health program. SOAR aims to increase the capacity of persons living with an increased risk of OA due to an activity-related knee injury to self-manage their knee health and knee OA risk. The program was developed alongside patient and clinician partners, and is based on past research, [10, 12, 24–26] clinical practice guidelines, [17] guidance for Developing and Evaluating Complex Interventions, [27] and is consistent with patient-centered care, [28] shared decision making [29] and behaviour change theory [30]. Recently we have established the feasibility of the SOAR program [31]. Prior to determining the effectiveness of SOAR it needs to be assessed in an ideal (efficacy) setting.

## Methods

### Aim

The primary objective of this study is to assess the efficacy of an 8-week SOAR program to improve knee extensor muscle strength, moderate-to-vigorous physical activity (MVPA), self-reported knee-related QoL, and knee-specific self-efficacy in people discharged from regular care after a sport or recreational-related knee trauma. Additional objectives are to evaluate the feasibility of a future hybrid effectiveness-implementation randomized controlled trial (RCT) protocol, determine the optimal intervention length, and explore stakeholder experiences.

### Study design and setting

This is a proof-of-concept, two-armed, open-label, randomized delayed control trial [32] with embedded 1:1 interviews. In this design, randomization determines when the intervention is provided (immediate or 9-week delay). A delayed-control is appropriate as the standard of care after discharge from knee trauma care is no care, and the intervention is beneficial and low risk [33, 34].

The study is guided by the Standard Protocol Items: Recommendations for Intervention Trials, [35] and Standards for Reporting Qualitative Research [36]. Protocol reporting follows the Standard Protocol Items: Recommendations for Intervention Trials, while trial reporting will follow the Consolidated Standards of Reporting Trials statement [37] and Consensus on Exercise Reporting Template [38]. Protocol feasibility assessments will be guided by Bowen et al. [39] and Thabane et al. [32] The research will be conducted at the University of British Columbia (UBC) and Arthritis Research Canada, in Vancouver, Canada between December 2021 and December 2022. The study is approved by the UBC Clinical Research Ethics Board (REB #H21–01491) and all participants will provide informed consent and complete a Physical Activity Readiness questionnaire (PAR-Q,2002) prior to testing [40].

### Participants

Both persons who have experienced a past sport or recreational-related knee injury and physical therapists will be recruited during this study. Knee injury participants will include a convenience sample of individuals who are at least 12-, but not more that 48-months, past an activity-related knee injury, 16–35 years of age, and not receiving on-going healthcare for their knee. A 12–48-month post-injury period is consistent with completion of knee injury rehabilitation (typically ≤12-months), [41] precedes radiographic OA, [4] and has been identified by patient partners as an opportune time. Activity-related knee injury is defined as self-reported knee trauma requiring medical consult that disrupted activity participation on more than one occasion [42]. Persons will be excluded if they have OA illness (i.e., movement related joint pain + morning stiffness < 30 min + functional limits with either crepitus, or motion loss per the EULAR criteria) [43]; inflammatory arthritis or systemic condition; leg injury, surgery, or injection in the past 6-months; pregnancy; no email address or daily access to a computer with internet; or refuse to wear an activity tracker.

Musculoskeletal PTs will also be enrolled to deliver the intervention to participants with a past knee injury. PT participants must be registered to practice in the province of British Columbia, Canada, able to communicate in English, be willing to complete Brief Action Planning (BAP) and SOAR program training, and have daily access to a computer with internet service.

### Recruitment

Participants with a previous knee injury will be recruited through the study’s social media accounts and established local PT clinics and sport organizations. PTs will be recruited through the British Columbia physical therapy research Collaboration Registry go.library.ubc.ca/9852gp and through word of mouth.

### Sample size

The sample size of knee injured participants is based on detecting a meaningful change in normalized peak knee extensor torque (Nm/kg) between study groups over 8-weeks, with 25% attrition. Bodkin et al. [44] reports that a gain in normalized knee extensor torque of 0.22 Nm/kg [44] discriminates people who achieve clinically relevant gains in self-reported knee function, 6–9 months post-trauma. To detect a mean change of 0.22 Nm/kg in the immediate intervention study group assuming no change in the delayed intervention study group, and a common standard deviation (0.25 Nm/kg), [11] a sample of 42 participants (21/group) is needed (two-sided test, 1-β = 0.8, α =0.05). Recruitment of 70 participants (35/group) allows for 25% attrition.

### Procedures

**Fig. 1:**
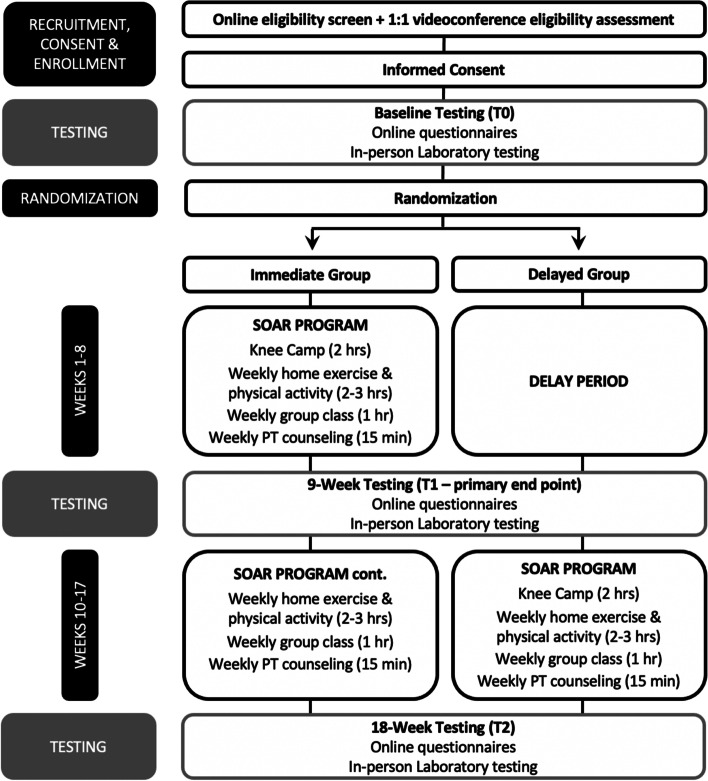
Overview of knee injury participant flow through the study

Figure 1 outlines the study phases for participants with a past knee injury. Interested persons will be directed to an online screening survey (Qualtrics XM, US) and those potentially eligible will be emailed study information and scheduled for a videoconferencing interview to confirm eligibility. Eligible persons will receive an URL to an electronic consent form located on a data management platform (REDCap 10.9.4, Vanderbilt University, US). After consenting, participants will complete baseline testing and be randomly allocated to study group (see below). The immediate study group (IG) will be complete an 8-week (weeks 1–8) SOAR program (Knee Camp, home-based exercise-therapy and physical activity with tracking, weekly 1:1 PT counseling sessions, and optional weekly group-based exercise classes) followed by an additional 8 weeks (weeks 10–17) of home-based exercise-therapy and physical activity with tracking, weekly 1:1 PT counseling sessions, and optional weekly group-based exercise classes. In contrast, after a 10-week delay, the delayed study group (DG) will complete an 8-week (weeks 10–17) SOAR program (Knee Camp, home-based exercise-therapy and physical activity with tracking, weekly 1:1 PT counseling sessions, and optional weekly group-based exercise classes). Knee injury participant outcomes will be evaluated at baseline (T0), 9-weeks (T1-primary endpoint to assess efficacy), and 18-weeks (T2- endpoint to inform optimal intervention length).

**Fig. 2:**
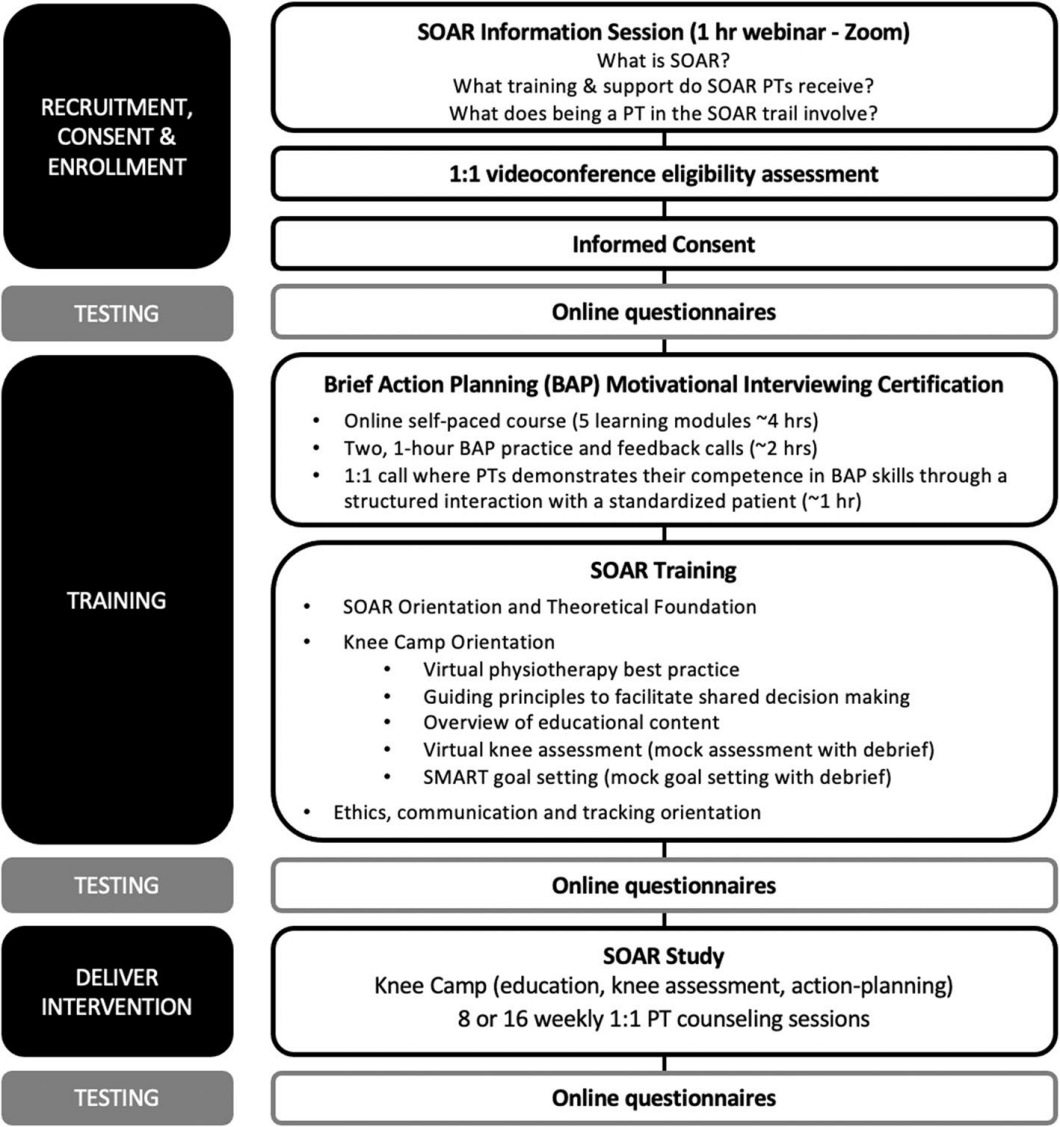
Overview of physical therapist participant flow through the study

Figure 2 outlines the study phases for the PT participants. Interested PTs will attend an online information session and then send an email to research team to express their interest. After a videoconferencing interview to confirm eligibility PTs will receive an URL to an electronic consent form located on a data management platform (REDCap 10.9.4, Vanderbilt University, US). After consenting, PT will complete baseline testing (online questionnaires), training and then will be randomly assigned to knee injury participants (see below). PT outcomes will be evaluated prior to training (baseline) immediately after training, and after they have delivered the SOAR program to all knee injury participants that they have been assigned in the study.

### Data collection and management

Data will be obtained through questionnaires hosted on a secure online survey platform (REDCap 10.9.4, Vanderbilt University, US). At each testing period knee injury participants will also attend a 1-h in-person laboratory session to complete computerized dynamometry testing of knee extensor and flexor muscle function, and a dual x-ray absorptiometry scan to estimate body mass index and body composition. At the end of the in-person session, participants will be given a triaxial accelerometer and instructed on how to wear it for 7-days. The accelerometer will be returned in a postage paid courier envelope. Secure online forms (Microsoft© Sharepoint) will be used to track knee injury participants attendance, adverse events, healthcare use, exercise/activity goal completion, Rating of Perceived Effort, and any associated pain. Data will be stored in secure electronic databases and de-identified. All authors will have access to the final dataset.

### Randomization allocation, concealment and blinding

After baseline testing, knee injured participants will be randomly assigned to IG or 10-week DG groups in a 1:1 allocation ratio, stratified on sex in variable block sizes [4, 6, 8]. A research coordinator will use serial labelled opaque envelopes prepared by a statistician (blind to data collection and daily trial activities) with a randomization schedule (SAS v9.4) to reveal group assignment.

The nature of the intervention does not allow for full blinding (i.e., participants and PTs cannot be blinded to study group). To reduce allocation bias, participants will be randomized to study groups by a research coordinator using serial labelled opaque envelopes prepared by our statistician (no interaction with participants and blind to data collection and daily trial activities). To reduce confirmation bias, persons leading outcome assessment and analyses will be blinded to group allocation, and participants will be asked not to disclose allocation to assessors. All questionnaires are self-reported (assessor blinded). Participants will also be blinded to study hypotheses.

### Intervention

The intervention is a virtually-delivered PT-guided knee health program called SOAR (Stop OsteoARthritis). The program has 3 components: 1) one-time Knee Camp; 2) individualized weekly home-based exercise-therapy, physical activity and tracking, and; 3) weekly 1:1 PT-guided exercise-therapy and activity action-planning with optional online group exercise class. Before starting, participants will recieve a Fitbit Inspire® activity tracker (Google LLC), workbook (educational materials), and resistance loop set (Chimaera®) providing up to 100 pounds of resistance to enable exercise progression.

#### Knee camp

This two-hour session, conducted over videoconferencing (Zoom®) includes a 1-h interactive group-based education session, 1:1 knee exam and exercise-therapy and physical activity goal-setting with a PT. The education session covers topics approved by patient partners, consistent with shared decision making theory, [29] clinical guidelines [41] and current understanding of OA (Table 1, Supplementary File 1 - Education session content). These concepts will be reinforced across the program. During the knee exam, PTs and participants co-identify and prioritize functional limitations. Exercise-therapy and activity goal-setting followed a Brief Action Planning (BAP) [23] approach (Fig. 1, Supplementary File 1 - BAP overview). Briefly, PT’s guide participants to identify at least one individualized home-based exercise-therapy and one physical activity SMART (*specific*, *measurable*, *attainable*, *relevant*, and *time-bound*) goal with tasks and adequate dose (target Rating of Perceived Effort [45] to address their unique functional limits (Table 2, Supplementary File 1 - Exemplar SMART Goals) for week 1. Participants can use the resistance band kit, body weight, common household materials (i.e., furniture, stairs) or any exercise equipment that they had access to when developing their dose. Goals will be modified until participant’s confidence to execute them rates ≥ 7/10. Actions to address perceived barriers will also be discussed. Participants will be instructed to wear it 24-h/day and share their Fitbit® activity ‘Dashboard’ with researchers. Finally, participants will be orientated to a Participant-Tracking form, where they will record their week 1 exercise-therapy and physical activity goals.

#### Weekly home-based exercise-therapy, physical activity and activity tracking

At home, participants will work to meet their exercise-therapy and physical activity goals. Degree of exercise-therapy goal completion, Rating of Perceived Effort, and any associated pain will be recorded on the Participant-Tracking form, and physical activity (Fitbit®) data synchronized with the Fitbit® online ‘Dashboard’.

#### Weekly PT-guided exercise-therapy and physical activity action-planning

Each week knee injury participants will attend a short (~ 15–30) 1:1 virtual PT counseling session and have the option of supplementing their home program with a regularly scheduled one-hour virtual PT-guided group exercise class (Table 3, Supplementary File 1 - Group exercise class menu). At the weekly counselling sessions PTs will ask and record responses to questions related to adverse events, medication and healthcare use, Fitbit® wear, and SMART goal completion on a bespoke PT tracking form. Participants and their PT will progressively modify or add SMART exercise-therapy and physical activity goals (using a BAP approach) based on the past weeks goal completion, physical activity (Fitbit® Dashboard), symptoms and obstacles encountered. At group class, participants will receive added instruction and feedback about exercise performance and progression. Participants will be able to email their PT between sessions as needed.

### Physiotherapist training

Prior to delivering the intervention, registered musculoskeletal PTs will complete BAP training and certification (8 h), and SOAR training (6 h) (Table 4, Supplementary File 1 – SOAR PT Training). BAP skills training and certification will be conducted in coordination with the Centre for Collaboration, Motivation and Innovation (Canada). BAP Certification (1-h role-play scenario) will follow a 4-h online course and three, 1-h group practice and feedback sessions. During the SOAR training, PTs will be oriented to the theoretical foundations underpinning the intervention, Knee Camp educational content, and observe and practice a virtual knee exam and SMART goal setting with a simulated patient followed by a group debrief. PTs will also be instructed in how use Zoom®, best-practice for virtual rehabilitation, [46] communication with knee injury participants, research ethics principles and how use an online PT Tracking form.

### Outcomes

Table 1 outlines study outcomes and measurement timepoints. The primary and secondary outcome choices are based on feasibility study data, [31] considered relevant by patient partners, and reliable and valid. Exploratory outcomes will be collected to assist in optimizing the effect and length of the intervention and determine which components are essential for efficacy and those that can be adapted for implementation.

**Table 1:**
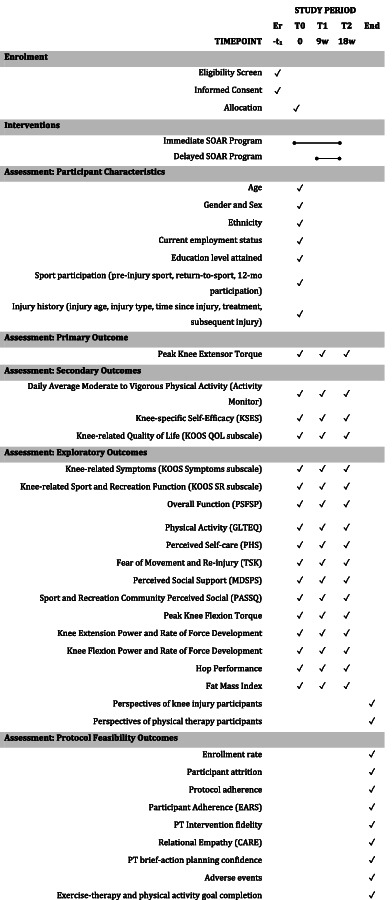
Schedule of Enrolment, Interventions and Assessments

Er (enrollment), T0 (allocation), T1 (primary end point), T2 (end point to inform optimal intervention length), End (end of study)

*CARE* Consultation and Relational Empathy Measure, *EARS* Exercise Adherence Rating Scale, *GLTEQ* Godin Leisure Time Exercise Questionnaire, *KOOS* Knee injury and Osteoarthritis Outcome Score, *MDSPS* Multi-Dimensional Scale of Perceived Support Scale, *mo* month, *PASSQ* Perceived Available Social Support Questionnaire, *PHS* Partner and Health Scale, *QOL* Quality of Life, *SR* Sport and Recreation, *TSK* Tampa Scale of Kinesiophobia

Protocol feasibility outcomes will be collected to inform the design of a future hybrid effectiveness-implementation RCT.

#### Participant characteristics

Age, gender (woman, man, gender-diverse, trans-gender, other), sex (female, male, intersex), ethnicity, current employment status, education attained, sport participation (main pre-injury sport, return-to-sport, participation in last year), and injury history (injury age, injury type, time since injury, treatment received, subsequent injury) will be collected with a baseline questionnaire. Participants were asked to answer all questions in reference to the study knee injury, or in the case of bilateral knee injuries, the most symptomatic.

#### Primary outcome

##### 9-week change in normalized peak knee extension torque

Quadriceps weakness is an established independent risk factor for OA illness, [47] and deficits in quadriceps function persist for years beyond knee trauma [11, 48]. In a recent meta-analysis quadriceps weakness was associated with increased odds of symptomatic knee OA in women (OR 1.85, 95%CI 1.29,2.64) and men (1.43, 1.14,1.78) [49]. Isokinetic concentric and eccentric knee extension torque will be assessed on computerized dynamometer (Biodex, System 4™, Biodex Medical Systems Inc. USA) at 60 degrees/sec over 0–100 degrees [50]. The peak torque reached over 3 repetitions will be recorded and normalized to body mass (Nm/kg).

#### Secondary outcomes

##### 9-week change in average daily MVPA

Inactivity increases OA risk [18] and physical activity attenuates adiposity 3–10 years after knee injury [10]. Average daily MVPA physical activity will be assessed with a waist-worn triaxial accelerometer (Actigraph™ GT3XP, Actigraph USA). Participants will be asked to wear the device in the correct orientation over the right anterior superior iliac spine (waist-worn) for a period of 7-continuous days, only removing for water/bathing or activities that may damage it. Participants will complete a monitor wear log to record non-wear times, including the duration and intensity (i.e., light, moderate, or vigorous) of activities performed when the device was not worn. Physical activity data will be included if the wear period exceeded ≥5 days (including at least one weekend day), with ≥10 h of data recorded per day. Using ActiLife™ software (version 6.1), raw acquisition data will be extracted in 10-s epochs using the Low Frequency Extension parameter. Wear time will be validated using the Choi algorithm (2011) [51] and cross-referenced with the self-reported monitor wear-log, and the Troiano algorithm^21^ will be used to categorize MVPA.

##### 9-week change in self-reported knee-related self-efficacy

Self-efficacy, or one’s belief in their ability to organize and execute actions to manage a prospective situation, [52, 53] predicts health behaviour including exercise participation [54]. The 19-item Knee Self-efficacy Scale (KSES) [55] will be used to measure self-reported knee-specific self-efficacy. Each item is scored on an 0–10-point Likert scale, with 0 indicating no knee-related self-efficacy and 10 indicating full knee-related self-efficacy. Individual item scores are summed and divided by 19 to produce a total score ranging from 0 to 10 (0 representing poor knee-related self-efficacy and 10 representing full knee-related self-efficacy).

##### 9-week change in self-reported knee-related QoL

Patient partners identify QoL as the most relevant outcome and people with an activity-related knee injury face reduced QoL for up to 10-years [11]. The 5-item QoL subscale of the Knee Injury and Osteoarthritis Outcome Score (KOOS) questionnaire will be used to measure self-reported knee-related QoL. Each item is scored on an 0–10-point Likert scale, with 0 indicating no knee-related QoL and 10 indicating full knee-related QOL. Individual item scores are summed and transformed to a score out of 100 (0 representing poor knee-related QoL and 100 representing full knee-related QoL).

#### Exploratory outcomes

In addition to the outcomes listed below, the 18-week change in primary, secondary and exploratory outcomes will be measured in participants allocated to the IG to inform optimal intervention length.

##### 9-week change in self-reported knee-related pain, symptoms and function in sport and recreation

The 8-,18- and 5-item symptom, pain, function in sport and recreation subscales of the KOOS questionnaire will be used to measure self-reported knee-related pain, symptoms and function in sport and recreation [56]. Each item is scored on an 0–10-point Likert scale, with 0 indicating many symptoms and 10 indicating no symptoms. Individual item scores are summed to produce a total score. Higher scores indicate lower levels of knee-related pain and symptoms, and higher levels of knee-related sport and recreation function.

##### 9-week change in self-reported patient-specific overall function

The 3-item Patient Specific Functional Scale will be used to identify, quantify and assess changes in functional limitations that are most relevant to participants [57]. This scale prompts participants to identify three activities important to them and rate their ability to perform each activity on a 10-point numerical rating scale. Individual scale scores are summed and transformed to a 0–100 scale with higher scores indicating better outcomes.

##### 9-week change in self-reported physical activity

The 4-item Godin Leisure Time Questionnaire will be used to measure self-reported physical activity [58]. Using the number of 15-min bouts of mild, moderate, and strenuous physical activity a participant engages in over a typical seven-day period weekly metabolic equivalents of physical activity are calculated.

##### 9-week change in self-reported perceived self-care

The 12-item Partner in Health Scale will be used to measure perceived self-care (active involvement to self-manage an ongoing condition) [59]. Each item is scored on a 9-point scale. Scores on individual items are summed to produce a total score with lower scores indicating better ability to manage their knee health.

##### 9-week change in self-reported knee-related fear of movement and re-injury

The 11-item Tampa Scale of Kinesiophobia will be used to measure self-reported fear of movement and re-injury [60]. Each item is scored on a Likert-scale from strongly disagree to strongly agree. The item scores are summed to produce a total score with a high value indicating a high degree of fear.

##### 9-week change in self-reported perceived social support

Perceived social support is an important predictor of positive health outcomes in rehabilitation, [61] and has been linked to improved psychological (e.g., self-confidence), [62–64] and behavioral outcomes (e.g., exercise therapy adherence) [64] after sport-related knee trauma. The Multi-Dimensional Scale of Perceived Support is a 12-item instrument with three subscales designed to measure self-reported perceived support from family, friends, and significant others in general populations [65]. Each item is scored on a 7-point scale. Individual item scores are summed to produce a total score with higher scores indicating higher levels of perceived support. The 16-item Perceived Available Social Support Questionnaire will be used to assess perceived support from the sporting or recreational community [66]. This instrument measures availability of support across four subscales (i.e., emotional, esteem, informational, tangible). Each item is scored on a 5-point scale. Individual item scores are summed to produce a score for each subscale with higher scores indicating higher levels of perceived support.

##### 9-week change in normalized peak knee flexor torque

Hamstring weakness is common after knee trauma, [48] and compounded when the Semitendinosus tendon is used as a graft site for an Anterior Cruciate Ligament (ACL) reconstruction [67, 68]. Isokinetic concentric and eccentric knee flexion torque will be assessed on computerized dynamometer (Biodex, System 4™, Biodex Medical Systems Inc. USA) at 60^o^/sec over 0-100^o^ [50]. The peak torque reached over 3 repetitions will be recorded and normalized to body mass (Nm/kg).

##### 9-week change in normalized peak knee extensor and flexor rate of force development and power

Thigh muscle rate of force development and power have been overlooked components of muscle function, [48] despite compelling emerging evidence that they may be more strongly associated with knee function and symptoms early after injury [69–71] and in persons with OA [72]. Computerized dynamometer (Biodex, System 4™, Biodex Medical Systems Inc. USA) will be used to assess and calculate normalized knee extension and flexion rate of force development (Nm/s) and power (Watts). After completing the protocol for normalized peak concentric knee extension and flexion torque the participants’ knee joint will be fixed in 60 degrees of sagittal plane flexion. The peak values reached over three, 5 s repetitions will be recorded and normalized to body mass (Nm/kg).

##### 9-week change in hop performance

Hop testing is the most commonly used clinical assessment of knee-related functional performance in individuals following knee trauma [73]. The 6-m timed hop is a functional task that challenges dynamic knee stability, [74] and is associated with knee OA 5 years after knee trauma [75]. Participants hop forward with the goal of covering a 6-m distance as quickly as they can. Two practice trials will be completed on each limb, starting with the unaffected side to familiarize participants with the task. Participants will then complete two test trials on each limb, starting again with the unaffected side. The shortest time (seconds) taken to hop the 6-m distance will be recorded.

##### 9-week change in fat mass index

Individuals with a history of knee injury are 4.4 (95%CI 1.6,12.3) times more likely to be in the highest quartile of fat mass index (kg/m2) [10]. Fat mass index will be measured with Bioelectrical Impedance (Taninta Body Composition Analyzer, Model TBF-300A, Tanita Inc., USA) which is feasible method for assessing and tracking body composition in clinical settings. Participants will stand barefoot on the bioelectrical impedance platform during which the resistance to the flow of this single, high frequency alternating electrical current (500A at 50 kHz) will be measured. The device will be calibrated prior to each scan (according to the manufacturer’s protocol).

##### Perspectives of knee injury participants

At study end, knee injury participants will complete an online survey that will ask questions about their SOAR program experience (satisfaction, accessibility). Additionally semi-structured 1:1 interviews will be conducted with a purposive maximum variation (balanced by sex, age, group allocation, time since injury, adverse events and adherence) [76] sample of approximately 15–20 participants. Using an inductive approach and interview guide, interviewees will be asked open-ended queries about their experiences and perceptions of SOAR including self-management, social support and therapeutic relationship. Interview guides have been co-developed with patient partners, and will be piloted and refined during data collection to ensure developing themes are effectively illuminated. Probes and prompts will provide elaboration. Field-notes will be taken, and interviews recorded. Sampling will be informed by ongoing analyses [76]. Data collection will cease when no new themes are identified [77].

##### Perspectives of PT participants

At study end, PTs will complete an online survey of their SOAR program experience (satisfaction, accessibility). Additionally, semi-structured 1:1 interviews will be conducted with a purposive maximum variation (balanced by sex, age, years of practice) [76] sample of approximately 7 PTs. Using an inductive approach and interview guide. Interviewees will be asked open-ended queries about their experiences and perceptions of SOAR, including SOAR and BAP training, and possible implementation clinic processes. Interview guides have been co-developed with patient and PT partners, and will be piloted and refined during data collection to ensure developing themes are effectively illuminated. Probes and prompts will provide elaboration. Field-notes will be taken, and interviews recorded. Sampling will be informed by ongoing analyses [76]. Data collection will cease when no new themes are identified [77].

#### Protocol feasibility outcomes

Protocol feasibility will be assessed with implementation, practicality and acceptability outcomes [32, 39].

*Implementation* outcomes included enrollment rate, participant attrition (% of participants who withdrew or lost to follow-up), protocol adherence (% of intervention and assessment components completed), participant adherence (Exercise Adherence Rating Scale; EARS, Participant Numerical Rating Scale of Action Plan Completion), PT intervention fidelity (% of a 41-item checklist completed during one randomly recorded 1:1 knee camp and one weekly counselling sessions per PT; Table 5, Supplementary File 1 – Intervention Fidelity Checklist), relational empathy (Consultation and Relational Empathy measure; CARE) which is a key component of therapeutic relationship, and change in PT BAP confidence. The EARS is a six-item self-report tool for measuring adherence to home exercise. Scores range from 0 to 42, with higher scores indicating better adherence [78]. The CARE is a 10-item self-report tool that measures empathy in the context of the therapeutic relationship between a clinician and a patient. Scores range from 10 to 50, with higher scored reflecting more empathy [79].

*Protocol Practicality* outcomes included the number of self-reported adverse events (requiring medical treatment or medications, and/or interferes with function for two or more days directly related to SOAR) [80] over the course of the intervention, and exercise-therapy and physical activity goal completion (% of goals entirely, partially or not completed) tracked weekly at the PT counseling sessions.

### Statistical analyses

Descriptive statistics will be calculated for demographic and potential confounding variables (i.e., injury type, time since injury, prior or subsequent injury, prior treatment), and observed differences will be considered when interpreting findings, and during the design of a future hybrid effectiveness-implementation RCT. Randomization integrity will be monitored. Mean changes in all outcomes between T0 and T1, and T1 and T2 will be described by study group and sex (knee extension torque) or gender (physical activity, KOOS, KSES).

**Fig. 3:**
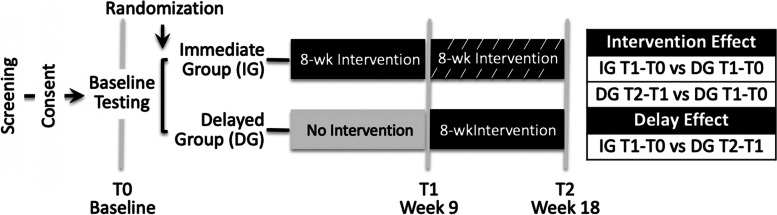
Analysis contrasts

We will conduct intent-to-treat analyses (compare outcomes according to randomized study groups, regardless of intervention adherence). Missing outcome data due to missed visits or dropouts will be handled using multiple imputations of missing values. Generalized linear mixed-effects regression models for longitudinal data (95%CI), controlling for blocking effect, will estimate the effect of the 8-week intervention (IG T1-T0 versus DG T1-T0, and DG T2-T1 versus T1-T0) and delay (IG T1-T0 versus DG T2-T1) for the primary outcome (knee extension torque change; Fig. 3).

We will conduct three exploratory analyses to guide design of the future hybrid effectiveness-implementation RCT. To inform primary outcome choice individual generalized linear mixed-effects regression models for longitudinal data (95%CI), controlled for blocking effect, will explore the effect of the 8-week program (IG T1-T0 versus DG T1-T0) on MVPA time, KOOS function in sport/recreation sub-scale, and KSES considering gender. To inform the most relevant muscle function outcome individual linear regression (95%CI) models, adjusted for age and sex, will compare the association between the change in knee extensor muscle function torque, power, and rate of force development over the 8-week intervention with the KOOS_4_. To inform optimal intervention length, a longitudinal mixed-effects model will examine the intervention effect at 18-weeks. This model will include the following fixed effects indicators: 1) study group (i.e., IG or DG) to account for baseline difference; 2) follow-up point (i.e., T1 or T2) to account for secular trend, and; 3) time since intervention initiation (i.e., 18- or 9-weeks) to estimate effects after these time intervals. The model will also include participant-specific random effects to account for repeated measures. As type II error is a greater concern in proof-of-concept trials than type I, we will not adjust for multiple comparisons [81].

Interview recordings will be transcribed verbatim and de-identified. Using a constant comparative approach [77] data will be coded and categories developed by comparing and determining meaningful patterns across codes. High-order themes will illuminate the relationship between categories. We will look for uniqueness in experience by gender. If gender-based themes are identified, data will be reanalyzed with a gender lens. Analysis credibility and trustworthiness will be fostered through data immersion, reflexive journaling, field notes, memoing and regular research team discussions of coding, early concepts and developing themes. A detailed audit of analytic decisions will be kept [82].

### Patient and clinician partner involvement

Three patient partners (young adult with lived experience of a sport-related ACL reconstruction, middle-aged adult with lived experience of a sport-related ACL reconstruction, re-injury, and recent knee OA diagnosis, and a middle-aged adult with lived experience of a sport-related ACL reconstruction, knee OA and knee arthroplasty), and three clinician partners (two PTs with 9 -years of clinical experience and one PT with 3-years) were engaged throughout the study. The patient and PT partners provided guidance on research objectives, appropriateness of outcomes, funding applications, and the development of the exit survey, SOAR participant workbook and Knee Camp content. They also participated in recruitment, and data analysis interpretation.

### Monitoring

Bi-weekly meetings between the research coordinator and the lead investigator will be held to monitor recruitment, adverse events, other problems and trial timelines. Regular contact between the research team and PTs will monitor problems associated with implementation of the intervention.

### Dissemination plans

The findings of the study will be presented at relevant scientific and professional conferences, published in relevant peer-reviewed journals and disseminated through the Arthritis Society, Canadian MSK Rehab Network and Versus Arthritis Center for Sport, Exercise and Osteoarthritis. Knee injury and PT participants will be provided with a lay summary of findings.

## Discussion

This trial will assess the efficacy of a novel, 8-week virtually-delivered, PT-guided knee health program to address OA risk factors and knee health in persons at increased risk of early-onset knee OA due to an activity-related knee injury. In addition, this study will provide invaluable data to inform optimal intervention length and a future Hybrid-1 Effectiveness and Implementation RCT. The relationships with patient, clinical and sport organizations fostered during this work will support future evaluation, implementation and scale-up. This research represents a first, vital step towards mitigating the consequences of activity-related knee injuries and the burden of OA.

## Supplementary Information

**Additional file 1.**

## Data Availability

Data sharing is not applicable to this article as no datasets were generated or analysed during the development of this protocol.

## Abbreviations

ACL: Anterior cruciate ligament
BAP: Brief action planning
CARE: Consultation and Relational Empathy measure
CI: Confidence interval
DG: Delayed intervention group
EARS: Exercise Adherence Rating Scale
EULAR: European League Against Rheumatism
IG: Immediate intervention group
KSES: Knee Self-Efficacy Scale
KOOS: Knee injury and Osteoarthritis Outcomes Score
MVPA: Moderate-to-vigorous physical activity
OA: Osteoarthritis
PAR-Q: Physical Activity Readiness Questionnaire
PT: Physical therapist
RCT: Randomized controlled trial
QoL: Quality-of-life
SAS: Statistical Analysis System
SMART: Specific, measurable, attainable, relevant, timebound
SOAR: Stop osteoarthritis
UBC: University of British Columbia
URL: Uniform resource allocator
