# Supplementary material for: Efficacy of the SOAR knee health program: protocol for a two-arm stepped-wedge randomized delayed-controlled trial
Source: BMC Musculoskelet Disord. 2022 Jan 25;23:85. doi: 10.1186/s12891-022-05019-z (PMC8790851; doi:10.1186/s12891-022-05019-z)
Supplement: Supplementary file 1 — Additional file 1. [file 12891_2022_5019_MOESM1_ESM.docx]

**SUPPLEMENTARY FILE 1: SOAR PROGRAM DETAILS**

**TABLE 1: Knee Camp Educational Content**

| **TOPIC** | **DETAILS** |
| --- | --- |
| - Define and explain condition | - Short and long-term consequences of knee trauma, poor knee health and OA - Define OA as a disease and discuss etiology, and pathology |
| - Identify risks and benefits of an intervention in relation to a condition and general health | - Benefits and risks of exercise and physical activity for knee health, OA and general health - Specific emphasis is given to modes of resistance (strength, endurance, neuromuscular control, power) exercises, cyclical weight-bearing exercises and aerobic activities |
| - Promote participants’ ability (confidence) and self-efficacy to act | - Explain ‘rate of perceived effort’ and ‘repetitions in reserve’ and how to use them to adjust exercise dose to ensure a therapeutic benefit. - Explain pain physiology and the role of pain in adjusting exercise dose and pacing |
| - Clarify participants values and preferences | - Participants are asked to reflect on what their main functional deficits are and their priorities for exercise-based activities. - Exercise and physical activity goals are co-developed in an individualized manner with values and preferences in mind |
| - Present current evidence on what is known and make recommendations | - Discuss current understanding and debunk any myths about OA, surgery and the role of weight bearing exercises and physical activities |
| - Check/clarify patient’s understanding | - Create a safe environment, provide time and facilitate participants questions and comments |
| - Arrange follow-up | - PTs and participants decide on the date and time of the next week’s PT counseling session prior to the end of Knee Camp. A calendar reminder and secure Zoom® link are sent |
| - Make or explicitly defer a decision | - Using Brief Action Planning approach participants and PT develop explicit specific, measurable, attainable, relevant, and time-bound (SMART) weekly exercise-therapy and physical activity goals. |

**FIGURE 1: Brief Action Planning Flowchart**

**Exemplar Application of Brief Action Planning in the Context of SOAR**

***First Session during Knee Camp***

1. *“Based on what you learned today, is there any exercise or physical activity that you would like to do to improve your knee health in the next week?”*
   1. If the participant shares an idea, help them make a SMART goal.

*“Many people have found it useful to get very specific about their plan. Would that work for you?”*

With permission, gather as many details as welcomed or helpful:

*“What?”* (specific exercise(s) or physical activity(s))

*“When?”* (time of the day and day(s) of the week)

*“Where?”* (physical location)

“How often/long/much” (sets and reps, load, times per week)

*“When would you like to start”* (day of the week)

- 1. If the participant wants or needs assistance, offer a behavioural (exercise or physical activity) menu.
     1. First, ask permission to share ideas.

*“Would you like me to share some ideas that others have used or that might fit for your situation?”*

- - 1. Suggest *2-3 therapeutic exercises* all at once without a pause. Ideas should be relevant to their goal. Use the last idea to prompt one of their own.

*“Some things you might try are ______, ______, or maybe something has come to mind now that I have shared a few examples. Do any ideas work for you?”*

- - 1. When an idea is chosen, help them make a SMART goal (see 1a above).
  1. After the participant has made a specific plan, elicit a commitment statement.

*“Just to make sure we’re on the same page, would you mind putting your plan together and saying it out loud?”*

1. *“Now that you have established a plan, I would like to know how confident you feel about carrying out your plan. Consider a scale of 0 to 10 where ‘0’ means you are not confident at all and ‘10’ means you are very confident – how confident are you about completing your plan?”*
   1. If confidence is ≥7, reaffirm their confidence and move on to establish a follow-up plan.

*“That’s great. It sounds like you have a good plan!”*

- 1. If confidence level is <7, problem solve to overcome barriers or adjust the plan.

*“__________ is great. It’s a lot higher than 0 and shows a lot of interest and commitment. We know that when confidence is a 7 or higher, people are more likely to complete their plan. Do you have any ideas about what might raise your confidence?”*

- 1. If the participant doesn’t have any ideas, ask if they would like suggestions.

*“Would you like to hear some ideas that might raise your confidence?”*

- 1. If they say yes, provide 2-3 ideas.
     *“Sometimes people cut back on their plan, change their plan, make a new plan, or decide not to make a plan. Do any of these ideas work for you or maybe you have an idea of your own?”*
  2. If the plan has changed, repeat 1c (commitment statement) and 2 (re-evaluate confidence).

1. *“Would it be useful to set up a follow-up to check on how you are doing with your new plan?”*

*“When”* (date, time of day)

*“How”* (Zoom)

*“With who”* (yourself, research assistant)

If they chose an exercise-therapy goal then repeat for a physical activity or vice versa.

***Follow-up***

1. *“How did it go with your plan?”*
   1. If they completed their plan, recognize and affirm their success.
   2. If they partially completed their plan, recognize and affirm their (partial) success.
   3. If they did not try their plan, say *“That’s okay! This is quite common when people are trying something new.”*
2. *“What would you like to do next?”*
   1. If the participant wants to make a new plan, start from the beginning of Brief Action Planning script.
   2. They may want to talk about what they learned from their plan. Reinforce learning and adopting the plan.
   3. If the participant doesn’t want to make another plan at this time, offer to return to action planning in the future.

| **TABLE 2: Examples of weekly individualized exercise therapy and physical activity goals** | | | | | | |
| --- | --- | --- | --- | --- | --- | --- |
| **I. Example Exercise-Therapy SMART Goals** | | | | | | |
| **Broad Objective** | **SMART GOAL** | | | | | |
|  | **Exercise** | **Dose** | **When** | **Where** | **Start date** | **Confidence** |
| Hike downhill for 5 km | Lunge | Reps: 15  Sets: 3/leg  RPE: 7/10*  Resistance: body weight | Time of day: after work  Day(s): M, W, F  Frequency: 3/ wk | Location: home | Oct 4 | 7/10 |
| Jump without pain | ¾ deep, 2-leg Squats | Reps: 8  Sets: 5  RPE: 8/10*  Resistance: 25lb band | Time of day: after work  Day(s): M, T, F  Frequency: 3/ wk | Location: home | Oct 4 | 8/10 |
| **I. Example Physical Activity SMART Goals** | | | | | | |
| **Broad Objective** | **SMART GOAL** | | | | | |
|  | **Activity** | **Dose** | **When** | **Where** | **Start date** | **Confidence** |
| Hike downhill for 5 km | Walking on undulating surface | Duration: 1 hr  Distance: 4km  RPE: 6/10** | Time of day: after breakfast  Day(s) of week: S  Frequency: 1/ wk | Location: Lynn Valley Ravine | Oct 9 | 7/10 |

*if the prescribed sets and repetitions of an exercise do not produce the prescribed perceived effort (RPE), participants will be instructed on how to add resistance OR increase the number of repetitions in each set until the prescribed perceived effort is reached.

** if the prescribed duration and distance of an activity does not produce the prescribed perceived effort (RPE), participants will be instructed on how to increase the duration OR distance covered in a set duration until the prescribed perceived effort is reached.

F (Friday), hr (hour), Km (kilometer), M (Monday) RPE (rating of perceived effort), T (Tuesday), W (Wednesday), wk (week)

**Group Exercise Class Exercise Menu**

Each week, participants can supplement their home program with an optional regularly scheduled virtual PT-guided group exercise class. This class consists of a 5-minute warm-up (exercises to physiologically prepare the musculoskeletal and cardiovascular systems for exercise), 45-minutes of vigorous exercises (7-8 Rating of Perceived Effort; RPE), and 5-minute cool-down. PTs help participants chose, or adapt, exercises from six groupings that target specific muscles with four progression levels, to accommodate their ability.

| **TABLE 3: Weekly Exercise Class (optional) Exercise Menu** |
| --- |
| **EXAMPLE DYNAMIC WARM-UP EXERCISES ~5 minutes** |

| **1 - FRONT PLANE** | | | |
| --- | --- | --- | --- |
| 1. High knees on the spot (march or jog) | 1. Forward high knee skip | 1. Forward high knee run | 1. Forward-backward high knee run |

| **2 - SIDE PLANE** | | | |
| --- | --- | --- | --- |
| 1. Lateral walk with resistance | 1. Lateral shuffle | 1. Lateral high knees | 1. Lateral hops or speed skaters |

| **3 - AGILITY** | | | |
| --- | --- | --- | --- |
| 1. Stationary quick feet | 1. Quick feet side step | 1. Stair/box toe taps (quick feet on stair) | 1. Quick front-back run with stutter steps |

| **4 - BALANCE** | | | |
| --- | --- | --- | --- |
| 1. Single leg balance | 1. Singe leg balance with ball toss | 1. Single leg triple extension | 1. Single leg pogo hops |

| **EXAMPLE EXERCISE PROGRAM EXERCISES ~45 minutes (Target RPE 7-8)** |
| --- |

| **1 - SQUAT PROGRESSION (Targets Gluteal, Quadriceps and Calf muscles)** | | | |
| --- | --- | --- | --- |
| 1. Sit to stand with heel raise | 1. Standing squat with heel raise | 1. Hands behind head standing squat with heel raise | 1. Standing squat with resistance and heel raise |

| **2- HAMSTRING PROGRESSION (Targets Hamstring muscles)** | | | |
| --- | --- | --- | --- |
| 1. Seated hamstring curl | 1. Deadlift | 1. Modified Nordic | 1. Full Nordic |

| **3 – SINGLE LEG PROGRESSION (Targets Gluteal, Quadriceps and Hamstring muscles)** | | | |
| --- | --- | --- | --- |
| 1. Stationary lunge | 1. Step-up | 1. Split lunge on step | 1. Single leg squat |

| **4 – ADDUCTOR PROGRESSION (Targets Groin and adductor muscles)** | | | |
| --- | --- | --- | --- |
| 1. Side lying bottom leg raise | 1. Standing adduction with resistance | 1. Copenhagen adduction exercise short lever | 1. Copenhagen adduction exercise with long lever |

| **5 – TRUNK PROGRESSION (Targets core muscles)** | | | |
| --- | --- | --- | --- |
| 1. Modified front plank   (from knees) | 1. Full front plank | 1. Full front plank saw | 1. Side plank |

| **6 – JUMP PROGRESSION (Targets Gluteal, Quadriceps and Calf muscles)** | | | |
| --- | --- | --- | --- |
| 1. 2-leg squat (snap downs) | 1. 2-leg counter movement jump | 1. 2-leg broad jump | 1. 1-leg jump |

| **EXAMPLE COOL DOWN ~5 minutes** |
| --- |

| Dynamic calf, quadriceps, hamstring, hip and trunk stretches |
| --- |

**SOAR Physiotherapy Training**

| **TABLE 4: SOAR Physiotherapy Training** | | |
| --- | --- | --- |
| **COMPONENT** | **DETAILS** | **LENGTH** |
| **I. BRIEF ACTION PLANNING (BAP) CERTIFICATION:** Done in coordination with the Centre for Collaboration, Motivation and Innovation (https://centrecmi.ca/) | | |
| - Online Asynchronous Self-paced Course | - 5 learning modules - 4 learning checks - Feedback survey | 3 hours |
| - BAP Practice and Feedback Calls | - 2, 1-hour small group calls with a BAP instructor where PTs have an opportunity to practice and get feedback on their BAP skills in a safe environment. | 2 hours |
| - BAP Certification Call | - 1:1 session where PTs demonstrate their competence in BAP skills through a strutured interaction with a standardized patient. | 1 hour |
| **II. SOAR DELIVERY WORKSHOP 1:** Hosted online by the SOAR research team | | |
| Introductions | | 0.5 hour |
| Orientation to SOAR & the spirit of SOAR | - Overivew of SOAR objectives, and components - Overview of guiding principles |  |
| Knee Camp (KC) Orientation | | 1.0 hours |
| Interactive Education Content | - Orientation to KC components - Orientation to educational content - Discussion about content and its role in setting the stage for the knee assessment and SMART goal development |  |
| Virtual Knee Assessment | - Virtual knee assessment orientation. - Demonstration of virtual knee exam (1:1) & charting - Practice virtual knee exam (1:1) & charting - Group debrief and problem solving - Next steps | 1.5 hours |
| **III. SOAR DELIVERY WORKSHOP 2:** Hosted online by the SOAR research team | | |
| Questions | - Questions from SOAR Delivery workshop 1 | 0.25 hours |
| Knee Camp Orientation cont.  Virtual SMART Goal Setting | - Adapting BAP to SOAR context and SMART goal setting - Demonstration of goal setting & PT tracking form completion - Practice goal setting & PT tracking form completion - Group debrief and problem solving - Adapting BAP to weekly 1:1 PT counselling sessions | 2.0 hours |
| Professionalism, Research Ethics, Communication & Resources | - Confidentiality - Discuss process for PT/participant communications, scheduling weeky conseling sessions - How to handle dropped communication - Discuss research team and web-based resources for PTs | 0.5 hours |
| Wrap-up Q & A | - Open question and discussion session - Overview of PT Roles | 0.25 hours |

**SOAR Intervention Fidelity Checklist**

**TABLE 5: Intervention Fidelity Checklist** (P = Performed; N = Not Performed; NA = Not Applicable)

| **Item** | **Description** | **P** | **N** | **NA** |
| --- | --- | --- | --- | --- |
| Knee Camp (1:1 Components other than BAP) | | | | |
| Introductions | Does the PT introduce themselves and provide space for the participant to introduce themselves? |  |  |  |
| Clarify Baseline Information | Does the PT mention and clarify the data they received from baseline testing? (name, pronouns, activities interests, age, index knee, injury, dominant leg) |  |  |  |
| Clarify Participant’s Goals | Does the PT confirm/clarify the participant’s stated Patient Specific Functional Scale goals and levels or identifies new goals as appropriate? |  |  |  |
| Explain Knee Exam | Does the PT explain the purpose and goal of the virtual knee exam? |  |  |  |
| Knee Alignment | Is standing stationary knee alignment assessed? |  |  |  |
| Knee Effusion | Is knee effusion and enlargement assessed? |  |  |  |
| Muscle Symmetry | Is between limb muscle symmetry assessed? |  |  |  |
| Crepitus & Palpation | Is knee joint crepitus and pain with palpation assessed? |  |  |  |
| ROM | Is bilateral knee joint ROM (flexion and extension) assessed? |  |  |  |
| Function | Is functional ability assessed through movement-related tasks? (e.g., squat, single leg balance, lunge, jumps, hops etc.)? If not, does the PT indicate why? |  |  |  |
| Virtual, 1:1 Counseling Session | | | | |
| Fit Bit Wear | Does the PT ask about last week Fitbit® wear/synchronization? |  |  |  |
| Adverse Events | Does the PT ask about adverse events (e.g., pain, swelling, activity)? |  |  |  |
| Medication | Does the PT ask about medication use in the last week? |  |  |  |
| Health Services | Does the PT ask about health service use in the last week? |  |  |  |
| Goal Completion | Does the PT ask about exercise and physical activity SMART goal completion? |  |  |  |
| Identified Barriers | Did the PT probe to identify exercise barriers and discuss with participants potential facilitators (as applicable)? |  |  |  |
| Brief Action Planning | | | | |
| Question 1 | PT clearly asks, “Is there anything you would like to do for your knee health...?” in an open-ended manner? |  |  |  |
| Skill 1: Behavioural Menu | Did the PT use a ‘Behavioural Menu’ when the person didn't have ideas, didn’t know where to start, or requested ideas? |  |  |  |
|  | If a ‘Behavioural Menu’ was used, did the PT asked permission to offer the ‘Behavioural Menu’? |  |  |  |
|  | Did the PT offer 2 or 3 brief and not specific ideas together in a list without pauses? Did the list have variety (i.e., not all one muscle group, not all one type of physical activity etc.) |  |  |  |
|  | Did the PT ask if the participant had ideas of their own as the last list item? |  |  |  |
| Skill 2: SMART Goals | Did the PT co-develop a SMART including (what, when, where, frequency, dose, intensity (PRE/RiR), duration, start date) IF the person was willing? |  |  |  |
| Skill 3: Commitment Statement | Did the PT ask the participant to verbalize their exercise and activities plan(s). |  |  |  |
| Question 2 | Did the PT ask the participant’s confidence level using a 0-10 scale, with a description of scale? (or a culturally appropriate alternative) |  |  |  |
|  | Did the PT respond positively to the self confidence level, and if the confidence level was < 7, explained the reason for a confidence level of ≥7? |  |  |  |
| Skill 4: Problem Solving Low Confidence | Did the PT use problem solving if confidence was < 7? |  |  |  |
|  | Did the PT ask for the participant’s own solutions first? |  |  |  |
|  | If the participant didn’t have their own ideas, did the PT offer a 3-part behavioural menu (see above)? |  |  |  |
|  | Did the PT ask for the commitment statement and confidence level again after the plan, if the plan was altered? |  |  |  |
| Question 3 | Did the PT and participant mutually agreed to a scheduled follow-up to check on participant's goal progress? |  |  |  |
| Skill 5: Progress Check | Did the progress check began with an open-ended question, “How did it go with your plan over the past week?” |  |  |  |
|  | Did the PT respond positively, no matter what the results were? |  |  |  |
|  | Did the PT ask an open-ended question to determine what the participant would adjust, and honoured the participants’ honoured? |  |  |  |
| Warmth and Tone | Did the PT use a warm and encouraging tone, while allowing the participant to do most of the talking? |  |  |  |
|  | Did the PT avoid language or statements that would reinforce their role as an ‘expert’? |  |  |  |
| Structure | Did the BAP items occurr in the order that they appear on this checklist? |  |  |  |
| Study Form Completion | | | | |
| PT Tracking Form | Did the PT complete all components of the PT tracking form for this encounter? |  |  |  |
| Protocol Deviations (based on the session being rated) | | | | |
| Confidentiality Breach | Did the PT breach participant confidentiality (e.g., include identifiers on forms, use personal email for communication, identify persons by full name in meetings with others, not obtained consent to record session if applicable)? |  |  |  |
| Ethical Violations | Did the PT engage in communication with the participant to facilitate a personal gain in any way (e.g., set-up an appointment with the participant outside the study, during or after study)? |  |  |  |
| Deviations from Study Protocol | Did the PT deviate from the study protocol? (e.g., meet with a participant twice in a week, prescribe exercise without co-development, encourage the participant to stop an activity when not contraindicated etc.) |  |  |  |
| Communication of Adverse Events | Did the PT promptly alert the SOAR study team of any potential severe adverse events? (as applicable) |  |  |  |
